# High prevalence of antibiotic resistance in commensal *Escherichia coli *among children in rural Vietnam

**DOI:** 10.1186/1471-2334-12-92

**Published:** 2012-04-18

**Authors:** Oliver James Dyar, Nguyen Quynh Hoa, Nguyen V Trung, Ho D Phuc, Mattias Larsson, Nguyen TK Chuc, Cecilia Stålsby Lundborg

**Affiliations:** 1 Division of Global Health (IHCAR), Department of Public Health Sciences, Karolinska Institutet, Nobels väg 9, 171 77 Stockholm, Sweden; 2 Health System Research, Hanoi Medical University, 1 Ton That Tung street, Hanoi, Vietnam; 3 Vietnam Cuba Friendship Hospital, 37 Hai Ba Trung street, Hanoi, Vietnam; 4 Department of Medical Microbiology, Hanoi Medical University, 1 Ton That Tung street, Hanoi, Vietnam; 5 Department of Probability and Statistics, Institute of Mathematics, 18 Hoang Quoc Viet road, Hanoi, Vietnam; 6 Oxford University Clinical Research Unit, National Hospital of Tropical Diseases, 78 Giai Phong road, Dong Da district, Hanoi, Vietnam

## Abstract

**Background:**

Commensal bacteria represent an important reservoir of antibiotic resistance genes. Few community-based studies of antibiotic resistance in commensal bacteria have been conducted in Southeast Asia. We investigated the prevalence of resistance in commensal *Escherichia coli *in preschool children in rural Vietnam, and factors associated with carriage of resistant bacteria.

**Methods:**

We tested isolates of *E. coli *from faecal samples of 818 children aged 6-60 months living in FilaBavi, a demographic surveillance site near Hanoi. Daily antibiotic use data was collected for participating children for three weeks prior to sampling and analysed with socioeconomic and demographic characteristics extracted from FilaBavi's re-census survey 2007. Descriptive statistics were generated, and a logistic regression model was used to identify contributions of the examined factors.

**Results:**

High prevalences of resistance were found to tetracycline (74%), co-trimoxazole (68%), ampicillin (65%), chloramphenicol (40%), and nalidixic acid (27%). Two isolates were resistant to ciprofloxacin. Sixty percent of isolates were resistant to three or more antibiotics. Recent sulphonamide use was associated with co-trimoxazole resistance [OR 3.2, 95% CI 1.8-5.7], and beta-lactam use with ampicillin resistance [OR 1.8, 95% CI 1.3-2.4]. Isolates from children aged 6-23 months were more likely to be resistant to ampicillin [OR 1.8, 95% CI 1.3-2.4] and co-trimoxazole [OR 1.5, 95% CI 1.1-2.0]. Associations were identified between geographical areas and tetracycline and ampicillin resistance.

**Conclusions:**

We present high prevalence of carriage of commensal *E. coli *resistant to commonly used antibiotics. The identified associations with recent antibiotic use, age, and geographical location might contribute to our understanding of carriage of antibiotic resistant commensal bacteria.

## Background

The growing problems posed by antibiotic resistance continue to be recognised and reported on [1-4]. Rising antibiotic resistance leads to increased mortality and morbidity, enhanced transmission of resistant bacteria, and increased health costs [2]. The European Centre for Disease Prevention and Control estimates that antibiotic resistance caused at least 25,000 deaths in Europe in 2007, which was over half the annual number of deaths caused by road traffic incidents in the same countries [5].

The mechanisms that produce antibiotic resistance are coded for in genes, which may be located on bacterial chromosomes or on mobile elements that may pass between different bacteria, such as plasmids [6]. An important reservoir of antibiotic resistance genes is found in commensal bacteria, which are further capable of facilitating the spread of such genes to pathogenic strains of bacteria [7,8]. The largest population of commensal bacteria is that found in the gut, where they are at risk of exposure to orally ingested antibiotics, as well as certain parenteral preparations [9].

Most studies describing the distribution of resistant bacteria have used clinical pathogens or commensal bacteria from people in healthcare settings. Data on resistance in commensal bacteria isolated from community settings have been reported on less frequently, especially so from Southeast Asia, where the findings have consistently shown high prevalences of resistance [9-12].

The most important determinant of the emergence of bacterial antibiotic resistance, both in individuals and populations, is antibiotic use [13]. In community settings young children tend to be the most exposed to antibiotics, and several studies have found that younger children have the highest risk of carrying resistant commensal bacteria [11,14]. *Escherichia coli*, a near ubiquitous coloniser of the gastrointestinal tract in children and adults, has often been used in studies of resistance in commensal bacteria [11,15-17]. *E. coli *has been shown to develop resistance in response to antibiotic use, and to be particularly capable of exchanging antibiotic resistance genes with pathogenic bacteria [7,18]. Recently, a number of other factors associated with carriage of resistant commensal organisms have been reported, but the various contributions of these remains unclear [15,16].

The aim of our study was to investigate the prevalence of resistance to antibiotics in commensal *E. coli *carried by preschool children in the population of a rural area of North Vietnam, and to further examine whether antibiotic use and a number of demographic and socioeconomic variables were associated with carriage of resistant *E. coli*. This study is part of a larger project conducted in a rural area in northern Vietnam where data have been collected on perceptions of child caregivers regarding antibiotic use [19], knowledge and practice of healthcare providers [20], prevalence of antibiotic resistance in *Streptococcus pneumoniae *[21] and reported antibiotic use [22].

## Materials and methods

### Study area

The study was carried out in Bavi district, 60 km west of Hanoi, Vietnam. The population of the region is approximately 250,000 persons, living in lowland, highland and mountainous areas. Children under five years of age account for 8% of the population. The public health care system consists of a 150 bed district hospital, 3 regional polyclinics and 32 commune health stations. There are also more than 200 private providers in the region, including private clinics, private pharmacies and traditional healers. The FilaBavi Epidemiological Field Laboratory was established in Bavi in 1998 [23]. Baseline and re-census surveys have been carried out every second year, with follow-up of vital events conducted every three months. FilaBavi is divided into units, referred to as clusters, with around 600-700 inhabitants in each cluster. In total there are 69 clusters within FilaBavi, including around 51,000 inhabitants and 12,000 households.

### Subjects and sample size

A sufficient number of clusters were included from each geographical area (lowland, highland, mountainous) to reach the total calculated sample size. This calculation was based on the finding from a previous study that 70% of children under five in the community used antibiotics during one month [24], with an expected drop out of 30%, and a design effect of 2.0 due to clustering. All of the households in the largest clusters within each geographical area were selected to include about 280 households in each area. Between March and June 2007 a total sample of 847 children was selected from 847 households in which at least one child was eligible for the study. One child aged 6-60 months at the time of study was randomly selected within each household that had more than one child fulfilling the inclusion criteria, using a computer. Data were extracted from FilaBavi's re-census survey 2007 on the participants' characteristics, date of birth, sex, residential areas, parents' education and households' assets, expenditure and income.

### Survey of drug use and sampling of isolates

Interviews with structured questionnaires were conducted weekly with the main child-caregiver in the household in order to collect information on reported daily illness symptoms and drug use for the participating child over a four-week period. Caregivers were requested to complete a form detailing daily drug use for the child. If available, the prescription or drug package was checked by the interviewers. Drugs used were classified according to the Anatomical Therapeutic Chemical (ATC) classification system [25] with the assistance of VN-pharmacy software [26]. This study includes only antibiotics classified as antibacterials for systemic use. These are aggregated at the level of the active ingredient (level 5 of the ATC class J01) [25].

Children were invited to attend the health commune station for a clinical examination on the 21st day of participation in the study. This was conducted by paediatricians from district or central hospitals. Faecal samples were collected by trained microbiologists, and immediately placed in a Cary-Blair transport tube. Within 12 h of sampling, all specimens reached the Clinical Laboratories of the National Institute of Infectious and Tropical Diseases, Hanoi.

*E. coli *was isolated and identified using standard laboratory procedures [27]. Briefly, presumptive *E. coli *isolates were picked based on typical colony morphology and Gram staining. Identification was confirmed by API 20E strip test (bioMérieux, France). *E. coli *serotyping was conducted following a protocol previously described [28], with the minor modification that the multiplex PCR assay was carried out on DNA extracted from a smear of bacteria from the first area of a MacConkey plate. Separate to this, a single colony was selected and sub-cultured for purity check and antibacterial susceptibility testing. The serotyping was conducted using a smear of colonies, whereas the antibiotic susceptibility testing used a single colony. Therefore it cannot be assumed that where diarrhoeagenic strains were found to be carried by a child that it was a colony of the same strain that also underwent susceptibility testing.

### Antibacterial susceptibility testing

For all isolates, inhibitory zone diameters were measured for tetracycline, co-trimoxazole, ampicillin, chloramphenicol and nalidixic acid using disk diffusion (BIO-RAD Laboratories, Marnes-la-Coquette, France). Minimum inhibitory concentrations were determined for ciprofloxacin using Etest (AB bioMérieux, Solna, Sweden, formerly AB BIODISK) for all isolates except for nine that did not survive storage. The antibiotics tested were those recommended for testing by the Clinical and Laboratory Standards Institute (CLSI) [29]. Susceptibility testing was carried out according to the performance standards of CLSI and the technical instructions of manufacturers. *E. coli ATCC 25922 *was used as the control strain in susceptibility tests. Testing conditions and reading of the diameter of the inhibitory zones and the MICs followed CLSI guidelines.

Interpretative breakpoints were based on the 2010 CLSI standards for this paper [30]. Susceptible or intermediately susceptible isolates were categorized as susceptible. Decreased susceptibility of isolates to the tested antibiotics was also determined using the epidemiological cut-off values (ECOFF) defined by the European Committee on Antimicrobial Susceptibility Testing (EUCAST) [31].

### Statistical analysis

Frequencies and proportions of isolates resistant to the tested antibiotics were calculated. A two stage process was used to determine associations with the outcome measures of (i) resistance to any antibiotic, and (ii) resistance to a specific antibiotic. The variables were first tested for association in univariate analysis using chi-square test, with those approaching statistical significance then entered into the binomial logistic regression model with backward selection. The independent variables assessed were: sex (male vs. female), age (6-23 months vs. 24-60 months), presence of diarrhoea (yes vs. no), economic status of the household (average or above vs. below average), number of children aged 0-17 living in household (one vs. more than one), area (lowland, highland vs. mountains), reported antibiotic use of any antibiotic class within the three weeks preceding the sample collection day (yes/no) and use of a specific class of antibiotic within the three weeks preceding the sample collection day (J01C, J01D, J01E, J01F). All statistical analyses were conducted using PASW Statistics 18.0 (SPSS Inc., Chicago, IL, USA), with a significance level of p = 0.05.

### Ethical considerations

Approval for the study was received from the ethical review board of Hanoi Medical University (No 28/HMURB, 2006). Permission for the study and use of census data was received from the FilaBavi steering committee. All parents were asked for verbal consent after explanation of the purpose and methods of the study. Confidentiality was assured, and participants were made aware of their right to withdraw at any time in the study period without explanation. Children identified at clinical examination as having need of medical care were treated and counselled by the paediatricians from the district or central hospitals.

## Results

### Study population

Of the 847 selected children, 818 (99%) caregivers consented for their child to participate in the clinical examination and collection of faecal samples. The characteristics of the children are shown in Table 1.

**Table 1:** Selected sociodemographic characteristics of participating rural children aged 6-60 months from FilaBavi, Vietnam

Characteristics		Participating children n = 818 (%)
**Sex**	Female	373 (46)
	Male	445 (54)
**Age**	6-23 months	367 (45)
	24-60 months	451 (55)
**Geographical area**	Lowland	268 (33)
	Highland	275 (34)
	Mountains	275 (34)
**Household economic status**	Below average	337 (46)
	Average or above	441 (54)
**Number of children in household**	One	219 (27)
	More than one	599 (73)
**Diarrhoea**	Present	10 (01)
	Not present	808 (99)
**Reported antibiotic use in 21 days** **before sampling**	Yes	477 (58)
	No	341 (42)

### E. coli serotypes

All 818 samples were analysed to detect the *E. coli *serotypes present. Non-diarrhoeagenic *E. coli *were found in 738 (90%) isolates. The most common diarrhoeagenic subtype was enterohaemorrhagic *E. coli *(EHEC), identified in 42 (5%) samples. Enteroaggregative *E. coli *was found in 28 (3%) samples, enterotoxigenic *E. Coli *in 26 (3%) samples, enteropathogenic *E. coli *in 12 (1%) samples, and enteroinvasive *E. coli *in one sample.

### Resistance to single and multiple antibiotics

**Figure 1:**
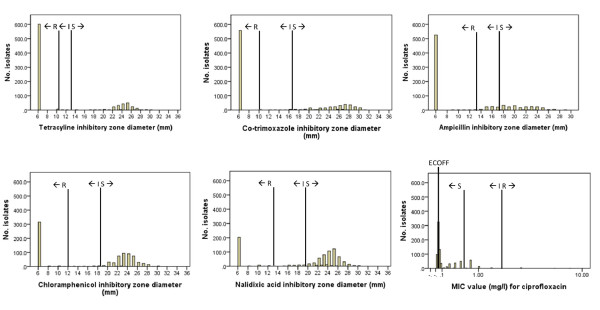
**Distribution of results from antibiotic susceptibility testing**. The results presented are based on data interpreted using CLSI clinical breakpoints. Abbreviations: S = Susceptible, I = Intermediately susceptible, R = Resistant (using CLSI clinical breakpoints); ECOFF = Epidemiological cut-off value breakpoint.

Only 46 isolates (6%) were sensitive to all antibiotics tested. Prevalences of resistance to single and multiple antibiotics are presented in Table 2. The distribution of zone diameter and MIC values are shown in Figure 1. Resistance to multiple antibiotics was very common, with 60% isolates resistant to three or more antibiotics tested. The modal value was resistance to four antibiotics. The most common patterns of resistance to multiple drugs were to tetracycline and co-trimoxazole (57% isolates), to co-trimoxazole and ampicillin (54% isolates), and to tetracycline and ampicillin (53% isolates). Combined resistance to tetracycline, co-trimoxazole, ampicillin and chloramphenicol was found in 25% isolates.

**Table 2:** Resistance prevalence to tested antibiotics among 818 isolates of *E.coli *from children aged 6-60 months in FilaBavi, Vietnam

Antibiotic(s) tested	Prevalence of resistance % (n, total n = 818)
TET	74 (609)
SXT	68 (559)
AMP	65 (533)
CHL	40 (325)
NAL	27 (220)
CIP	< 1 (2)
TET + SXT	57 (468)
TET + AMP	52 (424)
TET + CHL	34 (275)
TET + NAL	20 (164)
SXT + AMP	54 (441)
SXT + CHL	34 (277)
SXT + NAL	23 (185)
AMP + CHL	33 (270)
AMP + NAL	18 (151)
CHL + NAL	13 (109)
TET + SXT + AMP	45 (368)
TET + SXT + AMP + CHL	25 (208)
TET + SXT + AMP + CHL + NAL	8 (68)

Abbreviations used: TET = tetracycline; SXT = co-trimoxazole; AMP = ampicillin; CHL = chloramphenicol; NAL = nalidixic acid; CIP = ciprofloxacin

'+' indicates 'and'

The results presented are based on data interpreted using CLSI clinical breakpoints

### Determinants of resistance

Binomial logistic regression models were used to identify demographic, socioeconomic, and antibiotic use associations with resistance to any antibiotics tested, and to individual antibiotics (with the exception of ciprofloxacin due to the low number of isolates with ciprofloxacin resistance). Comparison prevalences for selected variables are shown in Table 3. Isolates taken from children aged 6-23 months were associated with resistance to ampicillin [OR 1.8, 95% CI 1.3-2.4], co-trimoxazole [OR 1.5, 95% CI 1.1-2.0], and chloramphenicol [OR 1.3, 95% CI 1.0-1.8]. Isolates from children living in the lowland area were less likely to be resistant to tetracycline [OR 0.62, 95% CI 0.42-0.90]. No association was found between antibiotic resistance and socioeconomic class, number of children living in the household, or presence of diarrhoea symptoms at time of sampling.

**Table 3:** Summary of resistance prevalence according to sociodemographic factors and antibiotic use among 818 isolates of *E.coli *from children aged 6-60 months in FilaBavi, Vietnam

	Prevalence of resistance %					
	AMP	SXT	TET	CHL	NAL	CIP
**Age**						
6-23 months	73*	73*	76	46*	30	0.3
24-60 months §	59	64	74	37	24	0.2
**Household economic status**						
Below average	67	68	78	40	24	0.3
Average and above §	64	68	72	41	29	0.2
**Geographical area**						
Lowland	60	66	68*	41	30	0.0
Highland	73	67	78	43	27	0.4
Mountains §	62	72	78	39	23	0.4
**Any reported antibiotic use in prior 21 days**						
No	56	63	73	37	23	0.6
Yes	72	72	76	44	30	0.0

*Result was statistically significant using binomial logistic regression

§Reference group used in binomial logistic regression

Abbreviations used: TET = tetracycline; SXT = co-trimoxazole; AMP = ampicillin; CHL = chloramphenicol; NAL = nalidixic acid; CIP = ciprofloxacin

The results presented are based on data interpreted using CLSI clinical breakpoints

Analysis of antibiotic use in the logistic regression models was restricted to the antibiotics most commonly used during the study period, beta-lactams, sulphonamides and antibiotics of the J01F class. Children that had taken sulphonamides were more likely to carry *E. coli *resistant to co-trimoxazole [OR 3.2, 95% CI 1.8-5.7], and resistant to nalidixic acid [OR 1.8, 95% CI 1.2-2.8]. Prior use of beta-lactam antibiotics was associated with carriage of *E. coli *resistant to any antibiotic tested [OR 2.3, 95% CI 1.3-4.0], resistant to ampicillin [OR 1.8, 95% CI 1.3-2.4], and resistant to chloramphenicol [OR 1.3, 95% CI 1.0-1.8]. Children that had taken antibiotics of the J01F class (macrolides, lincosamides and streptogramins) were more likely to carry *E. coli *that tested resistant to ampicillin [OR 2.1, 95% CI 1.1-4.1] and nalidixic acid [OR 1.7, 95% CI 1.0-3.0]. Children that used sulphonamides in the seven days prior to sampling only, or between eight and twenty-one days prior to sampling, had significantly higher carriage of *E. coli *isolates resistant to co-trimoxazole than children that did not take any sulphonamides during the study period (95% and 86% vs. 66%). Similarly, children that used beta-lactams exclusively in the seven days prior to sampling, or between eight and twenty-one days prior to sampling, were significantly more likely to carry isolates resistant to ampicillin (74% and 70% vs. 58%).

### Epidemiological cut-off analysis

For each antibiotic tested, zone diameters or MIC values were analysed using EUCAST epidemiological cut-off (ECOFF) values, in addition to using the clinical CLSI criteria. The ECOFF values identify bacteria that exhibit reduced susceptibility to antibiotics as compared with the wildtype population. Decreased susceptibility, according to ECOFF values, was found to tetracycline in 614 (75%) isolates, to ciprofloxacin in 605 (74%) isolates, to co-trimoxazole in 563 (69%) isolates, to ampicillin in 533 (65%) isolates, to chloramphenicol in 332 (41%) isolates, to nalidixic acid in 246 (30%) isolates. Eleven isolates (1.3%) showed wildtype susceptibility to all tested antibiotics. The MIC values for ciprofloxacin susceptibility testing are shown along with the ECOFF and CLSI clinical breakpoints for ciprofloxacin in Figure 1.

## Discussion

This is the first community-based study in Vietnam to explore the prevalence of antibiotic resistance and their determinants in commensal *E. coli*. It increases the limited knowledge of the prevalence of resistance to different antibiotics that are found in a rural population in a lower middle income country. A study of 113 children from rural areas around Hanoi conducted between 1996-1999 found similar resistance prevalences for co-trimoxazole, ampicillin and ciprofloxacin [10]. Comparisons between these studies are limited by differences in methodologies (we used CLSI interpretative breakpoints, whereas the authors in the previous study followed standards set by the Swedish Reference Group for antibiotics), and that the majority of children in this previous study had diarrhoea, whereas most children in our study did not (95% vs. 1% presence of diarrhoea).

A more recently published study in urban Hanoi examined resistance exclusively in diarrhoeagenic serotypes of *E. coli *in a mixture of children with diarrhoea in hospital and healthy children from daycare and healthcare centres, with no differences found in resistance prevalences between the two groups [32]. Compared with our study, that study reported higher prevalences of resistance to co-trimoxazole (89% vs. 68%), ampicillin (86% vs. 65%), chloramphenicol (77% vs. 40%), and ciprofloxacin (4% vs. < 1%), but a lower prevalence of resistance to nalidixic acid (19% vs. 27%). These differences may reflect higher levels of access and use of antibiotics in the urban setting. The prevalences identified in children in FilaBavi are considerably higher for most antibiotics than the few reported rural community studies in other areas of Southeast Asia, including Thailand and Indonesia [10,11]. Reports from community studies in rural areas in India and Peru have also presented lower prevalences of resistance in commensal *E. coli *than identified here [15,16].

Compared with children aged 24-60 months, isolates taken from children aged 6-23 months had higher prevalences of resistance to co-trimoxazole, ampicillin and chloramphenicol. This is in keeping with a few previous reports which have suggested that age can act as a risk factor for carriage of resistant bacteria, independent of increased antibiotic use of younger children [13,33]. The higher prevalence of ampicillin resistance in the highland area may be partly explained by a higher proportion of children taking beta lactam antibiotics in this area than in the other areas. Living in the lowland areas was independently associated with isolates having a lower prevalence of tetracycline resistance, a drug which should not be administered to children. This may reflect previously lower use of tetracyclines within the lowland area, or differences in exposure of *E. coli *to tetracylines from other sources, such as agriculture.

The majority of antibiotic use in our study was for the treatment of acute respiratory tract illnesses [22]. Analysis of specific antibiotic use showed that the antibiotics most commonly taken by the children, beta-lactams and sulphonamides, were strong risk factors for carriage of ampicillin and co-trimoxazole resistant isolates, respectively. This highlights the importance of individual antibiotic use in selecting for carriage of resistant *E. coli*. Some studies have failed to find associations between antibiotic use and resistance in commensals in community settings, which may reflect important differences in methodologies employed, such as not analysing by use of specific antibiotic classes [16,34,35]. Our data show a trend towards higher prevalence of co-trimoxazole and ampicillin resistance in isolates from children who took sulphonamide and beta-lactam antibiotics, respectively, in the seven days prior to sampling, compared with children who only took these antibiotics between eight and twenty-one days prior to sampling. Both of these subgroups remained significantly higher than children unexposed to these antibiotics during the study period.

CLSI criteria are used to identify bacteria that demonstrate resistance to antibiotics in a clinical setting, whereas ECOFF values are designed to identify bacteria that show reduced susceptibility to an antibiotic, as compared with the wildtype population of bacteria. Analysis of decreased susceptibility according to EUCAST ECOFF values produced comparable results for most antibiotics to those reached through the use of the CLSI clinical breakpoints. This reflects the similarity in the CLSI clinical breakpoint values and the EUCAST ECOFF values for *E. coli*. However, a considerable difference was observed for ciprofloxacin. Whereas 0.2% and 2.7% isolates were resistant or intermediately susceptible, respectively, using CLSI clinical breakpoints, 74% of isolates were found to have decreased susceptibility compared with the wildtype population, according to EUCAST ECOFF value. As few studies have thus far presented comparisons between the prevalence of decreased susceptibility using ECOFF values and the prevalence of clinical resistance using CLSI breakpoints, interpretation of this result must be made cautiously. It is possible that this represents an emerging decrease in susceptibility of *E. coli *in the study area to fluoroquinolones. A study by de Jong *et al. *also found significant differences between clinical resistance and decreased susceptibility to ciprofloxacin in commensal *E. coli *isolates from healthy food-producing animals in Europe, however the magnitude of the difference was less than that found in our study [36]. The EUCAST ECOFF value for ciprofloxacin in *E. coli *is based on 17877 observations [37], from which a similar definition of the wildtype population was reached using a normalized resistance interpretation method [38].

We identified a prevalence of EHEC carriage of 5%, which meant it was the dominant diarrhoeagenic *E. coli *subtype in our study. Two studies have found similar prevalences of EHEC carriage in asymptomatic individuals, one in workers from meat processing plants in Switzerland [39], and another in families living on dairy farms in Canada [40]. This high level of carriage might come from the large number of farm animals present in Bavi, or from other environmental sources, which we have not attempted to investigate in the present study. It may be that this high prevalence is due to a clonal spread, which could be explored in further studies. EHEC carriage was not associated with presence of any gastrointestinal symptoms, and its clinical significance remains unclear.

Our community study of resistance in commensal bacteria has several strengths, including its size and high response rate. The daily reporting of antibiotic use, validated by weekly follow up interviews, have enabled analysis of individual drug use to be correlated with antibiotic resistance, and suggested a trend towards higher resistance when antibiotics were consumed close to the date of sampling. There are also a number of limitations to our study. Importantly, only one *E. coli *isolate from each child was tested for antibiotic susceptibility, whilst it is appreciated that multiple *E. coli *strains usually co-exist in an individual's gastrointestinal tract [17,41]. The true prevalence of carriage of resistant *E. coli *is consequently likely to be higher than the figures presented here.

A further limitation is that the analysis on antibiotic use counted all days on which antibiotics were taken as equivalent, assuming that full doses had been taken. It has been noted elsewhere that sub-therapeutic dosing may be common in low- and middle-income countries [15]. Our study did not assess the contribution of certain potential exposures to antibiotics and antibiotic resistant bacteria remarked on elsewhere: antibiotic use by household members, day care attendance, exposure to contaminated water sources, contact with animal microflora. We have not investigated the prevalence of different mechanisms underlying the resistant phenotypes presented here.

## Conclusions

Our study has identified high prevalences of resistance to individual antibiotics in commensal bacteria isolated from children in a rural area of northern Vietnam. The high prevalences of resistance seen after individual antibiotic use highlight the increasing threat posed by antibiotic resistance in Vietnam, and suggest further evidence for the need for commitment to ensuring antibiotics are used in as rational a manner as possible.

## Competing interests

The authors declare that they have no competing interests.

## Authors' contributions

OJD, NQH, NVT, HDP, ML, NTKC, CSL participated in the conception and design of the study, data interpretation and revising paper critically for substantial intellectual content. NQH was responsible for the data collection and analysed data. OJD performed data analysis and drafted the manuscript. NVT was responsible for laboratory testing. NQH, NVT, HDP, ML, NTKC, CSL supported in the data collection and contributed to draft manuscript. HDP contributed to statistical analysis. All authors read and approved the final manuscript.

## Pre-publication history

The pre-publication history for this paper can be accessed here:

http://www.biomedcentral.com/1471-2334/12/92/prepub
